# Hyperpolarized ^131^Xe NMR spectroscopy

**DOI:** 10.1016/j.jmr.2010.10.004

**Published:** 2011-01

**Authors:** Karl F. Stupic, Zackary I. Cleveland, Galina E. Pavlovskaya, Thomas Meersmann

**Affiliations:** a Department of Chemistry, Colorado State University, Fort Collins, CO 80523, United States; b University of Nottingham, School of Clinical Sciences, Sir Peter Mansfield Magnetic Resonance Centre, Nottingham NG7 2RD, United Kingdom

**Keywords:** ^131^Xe, Xenon-131, Xe-131, Hyperpolarization, Hyperpolarized, Noble gases, Spin polarization, Spin-exchange optical pumping, Nuclear electric quadrupole moment, Quadrupolar relaxation, ^129^Xe, Nuclear magnetic resonance, Xenon-129, Krypton-83, ^83^Kr

## Abstract

Hyperpolarized (hp) ^131^Xe with up to 2.2% spin polarization (i.e., 5000-fold signal enhancement at 9.4 T) was obtained after separation from the rubidium vapor of the spin-exchange optical pumping (SEOP) process. The SEOP was applied for several minutes in a stopped-flow mode, and the fast, quadrupolar-driven *T*_1_ relaxation of this spin *I* = 3/2 noble gas isotope required a rapid subsequent rubidium removal and swift transfer into the high magnetic field region for NMR detection. Because of the xenon density dependent ^131^Xe quadrupolar relaxation in the gas phase, the SEOP polarization build-up exhibits an even more pronounced dependence on xenon partial pressure than that observed in ^129^Xe SEOP. ^131^Xe is the only stable noble gas isotope with a positive gyromagnetic ratio and shows therefore a different relative phase between hp signal and thermal signal compared to all other noble gases. The gas phase ^131^Xe NMR spectrum displays a surface and magnetic field dependent quadrupolar splitting that was found to have additional gas pressure and gas composition dependence. The splitting was reduced by the presence of water vapor that presumably influences xenon-surface interactions. The hp ^131^Xe spectrum shows differential line broadening, suggesting the presence of strong adsorption sites. Beyond hp ^131^Xe NMR spectroscopy studies, a general equation for the high temperature, thermal spin polarization, *P*, for spin I⩾1/2 nuclei is presented.

## 1 Introduction

In contrast to the extensive body of literature reporting nuclear magnetic resonance (NMR) studies with the spin *I* = 1/2 isotope ^129^Xe (110.5 MHz resonance frequency at 9.4 T, 26.4% natural abundance) [1–14], the only other NMR active stable isotope of this noble gas, ^131^Xe, has attracted much less attention since its first reported NMR observation in 1954 [15]. The ^131^Xe isotope (32.8 MHz resonance frequency at 9.4 T, 21.2% natural abundance) has a spin *I* = 3/2 and thus possesses a nuclear electric quadrupole moment (*Q* = −11.4 fm^2^) [16]. The electric quadrupole moment of the ^131^Xe nucleus is susceptible to interactions with electric field gradients (EFGs) and therefore serves as a sensitive probe for environmentally induced distortions of its large surrounding electron cloud [14]. Unless high concentrations of paramagnetic substances are present, these quadrupolar interactions are the dominant cause of ^131^Xe nuclear spin relaxation in all phases. Further, ^131^Xe coherent quadrupolar interactions can be induced when the xenon atoms are contained within an anisotropic environment.

### 1.1 ^131^Xe quadrupolar relaxation in solids, liquids and dissolved phase

In solid, natural abundance xenon, Warren and Norberg [17,18] found that ^131^Xe had a very short longitudinal relaxation time of *T*_1_ ≈ 200 ms at temperatures close to the melting point (161 K). However, the *T*_1_ increased monotonically by more than three orders of magnitude with decreasing temperature and reached *T*_1_ = 390 s at 9 K. The relaxation times in liquid xenon show the opposite trend compared to the solid and increase from *T*_1_ ≈ 40 ms at 161 K to *T*_1_ ≈ 80 ms at 250 K and 3 MPa. Later work [19] determined *T*_1_ = 110 ms at conditions just below the critical point, i.e. 298 K and 5.8 MPa. The ^131^Xe relaxation behavior of xenon dissolved in various solvents was subject to experimental and computational studies in the past (see [20] for a review). Longitudinal relaxation in polar solvents is quite fast (*T*_1_ < 10 ms) due to the electric field gradient fluctuations induced by the solvent molecule dipoles. Even in non-polar solvents, the ^131^Xe *T*_1_ relaxation times are typically below 50 ms.

### 1.2 ^131^Xe quadrupolar relaxation the gas phase

In gas phase, it was theoretically predicted by Staub and later confirmed experimentally by Brinkmann et al. [21] that the ^131^Xe longitudinal relaxation time (*T*_1_) is inversely proportional to the gas density, *ρ*, with

(1) $$1/T_{1}^{{}^{131}\text{Xe}}=\rho \cdot 3.96\times 10^{-2}\;{\text{amagat}}^{-1}\;{\text{s}}^{-1}\text{.}$$

1 amagat is the density of the specific gas at standard pressure and temperature of 101.325 kPa and 273.15 K. For xenon the atomic number density of one amagat is reported with 2.7048 × 10^25^ m^−3^ [22]. (Note that in literature the amagat is often alternatively defined as the density of an ideal gas at standard pressure and temperature resulting to the slightly different value of 2.6868 × 10^25^ m^−3^.) Brinkmann’s result was obtained at a temperature of 298 K and 0.76 T magnetic field strength. In later theoretical work, Adrian [23], considered separately the relaxation dependence on van der Waals and exchange contributions during binary collisions. He obtained $1/T_{1}^{{}^{131}\text{Xe}}=\rho \cdot 4.61\times 10^{-2}\;{\text{amagat}}^{-1}\;{\text{s}}^{-1}$ for the gas at room temperature but also noted a temperature dependence of the ^131^Xe relaxation. From these equations, a ^131^Xe gas-phase relaxation time of *T*_1_ ≈ 22–25 s would be expected at ambient pressure (∼1 amagat). However the equations above do not take into account ^131^Xe interactions with the surrounding container walls that accelerate the apparent gas-phase relaxation. The earlier works also do not consider relaxation caused by the formation of Xe–^131^Xe van der Waals complexes that leads to a gas density independent relaxation term [24–27] at the field strengths and gas pressures used in this work.

### 1.3 ^131^Xe quadrupolar coherence

Like the longitudinal relaxation, the spectral features observed in ^131^Xe NMR are dominated by this isotope’s high nuclear spin and large nuclear quadrupole moment. If ^131^Xe is placed in an anisotropic environment, for instance when dissolved in a liquid crystal, a triplet is observed in the NMR spectrum that displays resonance line splittings in the kHz regime. The triplet in liquid crystalline phase is caused by interactions of the nuclear quadrupole moment with the electric field gradient (EFG) induced by the anisotropic solvent (see [28] for a review). Even the surfaces of macroscopic containers can cause a ^131^Xe quadrupolar splitting that can be detected in the gas phase. This splitting was originally observed in spin-exchange optical pumping experiments at low magnetic fields of a few mG (see below) [29–35]. However, the effect of surface orientation and temperature on the gas phase ^131^Xe quadrupolar splitting can also be observed in thermally polarized high-field NMR spectroscopy [36,37].

### 1.4 Magnetic field induced ^131^Xe quadrupolar splitting

Another unique property of ^131^Xe is that a quadrupolar splitting pattern of a few Hz can also be generated in the bulk gas phase, independent of the presence of surfaces [19]. The effect is caused by high magnetic fields, ${\overset{\to }{B}}_{0}$, that generate an electric field gradient (EFG) in atoms located within this field. The EFG is a result of interactions of the external magnetic field ${\overset{\to }{B}}_{0}$ with the magnetization $\overset{\to }{M}$ of the xenon electron cloud. The EFG tensor orientation is always aligned with ${\overset{\to }{B}}_{0}$, thus leading to a quadrupolar splitting, reminiscent of the much stronger splittings in liquid crystals. As was shown previously with thermally polarized ^131^Xe [19], this “high-field’ quadrupolar splitting displays a quadratic dependence upon $\left|{\overset{\to }{B}}_{0}\right|$. Theoretical papers following the initial experimental observation agree with the quadratic magnetic field dependence of the splitting, but disagreed about the presence of an additional linear term [38,39]. At current, a magnetic field dependent splitting has only been observed with the noble gas isotope ^131^Xe, due to its unique combination of a large and easily distortable electron cloud, spherical symmetry of the unbound noble gas atoms, ‘high resolution grade’ NMR linewidth in the gas phase, and its large nuclear electric quadrupole moment at a relatively small spin *I* = 3/2 value.

### 1.5 ^131^Xe in materials

Quadrupolar interactions are highly sensitive to anisotropy of the local environment and solution-phase thermally polarized ^131^Xe NMR spectroscopy has been used to probe a variety of systems including liquid crystals contained in microchannels [40], bicelles [41], and macromolecules [42]. ^131^Xe NMR spectroscopy has even been applied to characterize xenon compounds [43,44]. Spectroscopic ^131^Xe studies of surfaces have also been performed at low temperatures [45] and in variety of porous materials [46–50]. Thermally polarized ^131^Xe magnetic resonance imaging (MRI) with liquefied xenon provided a contrast sensitive to surface adsorbed water in aerogels [51].

Unfortunately, the low gyromagnetic ratio and often kHz-broad linewidths of ^131^Xe lead to exceedingly small NMR signal-to-noise ratios when thermally polarized gas is used. As a result, the surface-specific insights provided by this isotope have primarily been confined to extremely high surface to volume ratio environments that generate rapid *T*_1_ relaxation or systems that can withstand xenon at high pressures. In contrast, the relatively long relaxation times observed in the gas phase and in the presence of low surface to volume materials make thermally polarized ^131^Xe NMR unpractical, in particular at low gas densities.

However, these conditions are ideal for studies employing hyperpolarized (hp) ^131^Xe that provides orders of magnitude of signal enhancement but also requires long relaxation times in order to preserve the hyperpolarization. Systems with longitudinal ^131^Xe relaxation times substantially shorter than *T*_1_ = 1 s do not permit meaningful applications of hyperpolarized ^131^Xe NMR, unless interfaces of theses systems to the bulk gas phase were to be studied.

### 1.6 Hyperpolarized (hp) ^131^Xe

Like all NMR active noble gas isotopes, high non-equilibrium nuclear spin polarization can be generated in gaseous ^131^Xe through alkali metal vapor spin-exchange optical pumping (SEOP) [52,53]. The fundamental details of hp ^131^Xe production have been explored in some detail by Volk [29,54], Happer [30–32], Pines [33], Mehring [34], and their respective co-workers. Luo et al. have also studied ^131^Xe SEOP using cesium in high magnetic fields at 11.7 T [55]. Optically detected NMR experiments using SEOP were applied in the past to study the influence of the glass container surfaces on the gas-phase hp ^131^Xe relaxation and were used to investigate xenon adsorption phenomena on glass surfaces [29–35]. The shape of macroscopic containers with centimeter-sized dimensions was found to cause an anisotropy in the effective electric field gradient that can lead to a small quadrupolar splitting, typically in the Hz regime or less. Following earlier work with ^201^Hg and ^83^Kr [56,57], the ^131^Xe splitting was observed at low magnetic fields in the gas phase contained in cylindrical cells [29–35]. The splitting was strongly dependent on the aspect ratio of the cell dimensions and the cell orientation within the magnetic field. However, this type of splitting was averaged out in cells with spherical symmetry if macroscopic gas diffusion allowed the xenon atoms to sample surface segments with different orientations during the relevant NMR timescale. In an intriguing experiment, Mehring and co-workers used optical detection of the hp ^131^Xe quadrupolar splitting in a rotating glass cell to construct a gyroscope that utilized geometric quantum-phase [58–60] (see Refs. [61,62] for further theoretical work). More recently, Kitching and co-workers studied the crossover regime between pure nuclear quadrupolar resonance and quadrupolar perturbed Zeeman effect at low magnetic field strengths [63] using optically detected hp ^131^Xe.

Previously, the hyperpolarized ^131^Xe was never separated form the reactive alkali metal vapor, thus limiting its application to non-reactive systems. The work presented here is concerned with the production of alkali metal free hp ^131^Xe and the peculiarities of ^131^Xe SEOP are explored. Transfer of the resulting hp ^131^Xe into high magnetic field NMR detectors enabled the study of the effects of gas composition and density on the spectral features and longitudinal relaxation of ^131^Xe. Additionally, the absence of alkali metal in the hp gas mixture was exploited to investigate the influence of surface adsorbed water vapor upon the ^131^Xe quadrupolar splitting and surface induced longitudinal relaxation. Finally, a general treatment of polarization and signal intensity observed hyperpolarized spin *I* > 1/2 nuclei is provided.

## 2 Experimental

### 2.1 Spin-exchange optical pumping (SEOP)

SEOP was carried out in a cylindrical Pyrex glass cell (length = 125 mm, inner diameter = 27 mm) containing 1–2 g of rubidium (99.75%; Alfa Aesar, Ward Hill, MA). The Pyrex glass cell was used without treatment of the internal glass surface due to fast quadrupolar relaxation of ^131^Xe on silane coated surfaces [31,64]. The highest spin polarization for ^131^Xe was obtained when the front end of the cell was kept at approximately 453 K while a temperature of 393 K proved to be best for ^129^Xe. The temperature was maintained through a flow of hot air that was temperature regulated by a controller monitoring the front of the SEOP cell that was approximately 5 K hotter than the back end of the cell. Illumination through the front window of the SEOP cell was provided by two 30 W COHERENT (Santa Clara, CA) continuous wave diode array solid-state lasers. Each laser delivered 20 W of 794.7 nm circularly polarized light after losses in the fiber optics and polarizing optics.

The duration of the stopped-flow SEOP was typically 5–10 min. This time period was longer than needed for the SEOP process itself but was required for equilibrium rubidium vapor pressure to recover after the shuttling procedure. The gas pressure in the pumping cell ranged from 120 kPa to 460 kPa, depending on the desired final pressure in the NMR detection cell. For the SEOP build-up experiments and for the relaxation measurements a pressure of 150 kPa was used. Hp gas was rapidly transferred into the NMR probe by pre-evacuating the detection cell to less than 0.1 kPa. The SEOP cell was then opened, and the gas pressures in the SEOP cell and the detection cell were allowed to equalize. Rubidium vapor was separated from the hp gas by an air-cooled filter, containing loosely packed glass wool located inside the transfer line between the pumping cell and the detection cell. The magnetic field necessary for optical pumping was provided either by the fringe field of the superconducting magnet (0.05 T for 9.4 T, 0.004 T for 11.7 T, and 0.04 T for 14.1 T) or by a Helmholtz coil pair (2.0 × 10^−3^ T).

### 2.2 Gas mixtures

Three gas mixtures used in this work were composed of naturally abundant, research grade gases provided by Airgas (Radnor, PA), with purities of 99.995% for Xe, 99.9997% for N_2_, and 99.9999% for He. The gas mixtures used in this study were 5% Xe, 5% N_2_, and 90% He (mixture I); 20% Xe, 5% N_2_, and 75% He (mixture II); and 93% Xe and 7% N_2_ (mixture III).

### 2.3 NMR measurements

High resolution ^131^Xe spectra were obtained at 11.7 T and 14.1 T, using a Varian INOVA 500 MHz spectrometer and a Chemagnetics Infinity 600 MHz spectrometer, respectively. Commercial 10 mm broadband probes tuned to ^131^Xe frequency at either field strength (41.23 MHz and 49.47 MHz at 11.7 T and 14.1 T, respectively) were used. The length of a *π*/2 pulse was 24.5 μs at 11.7 T and 35 μs at 14.1 T. The gas samples were shimmed using an external D_2_O standard for the field lock channel. The D_2_O was located between the walls of the outer tube (10.0 mm outer diameter, 9.1 mm inner diameter; Wilmad-LabGlass, Vineland, NJ) and a hp gas containing inner detection tube (custom-built medium wall NMR 8 mm outer diameter, 6 mm inner diameter, for 11.7 T; 5 mm outer diameter, 4.2 mm inner diameter for 14.1 T; Wilmad-LabGlass, Vineland, NJ).

Polarization build-up and relaxation data were collected at 9.4 T using a Chemagnetics CMX II 400 MHz spectrometer and a custom built probe tuned to the ^131^Xe frequency at 32.81 MHz. The length of *π*/2 pulse was 35 μs. A 15 mm outer diameter, 12.6 mm inner diameter Pyrex glass sample tube was used as sample holder. No resistive magnetic field shimming was provided because resolved quadrupolar lineshapes were not required for these experiments. *T*_1_ measurements were performed using a series of 16 equally spaced, medium flip angle (12.3°) radiofrequency (RF) pulses to probe the hp ^131^Xe polarization decay as a function of time.

To collect polarization build-up curves, SEOP cell conditions such as temperature, pressure, and illumination were initially maintained in the absence of magnetic field for 5–10 min to ensure that no non-equilibrium polarization was present. The magnetic field was then turned on for a period of time, *t_p_*, after which the hp ^131^Xe was delivered via pressure-equalization into the previously evacuated detection cell, and signal was acquired using a *π*/2 pulse. The signal enhancements for hp ^131^Xe were referenced to the thermal signal obtained from a sample containing only 810 kPa of natural abundance ^131^Xe. The applied 15 s recycle delay was sufficient to restore the full polarization after each pulse because the pure gas-phase relaxation time *T*_1_ for ^131^Xe at 810 kPa, obtained through Eq. (1), is approximately 3 s and is further reduced by interactions with the glass container wall and the formation of van der Waals complexes.

### 2.4 Co-adsorbing water experiments

**Fig. 1:**
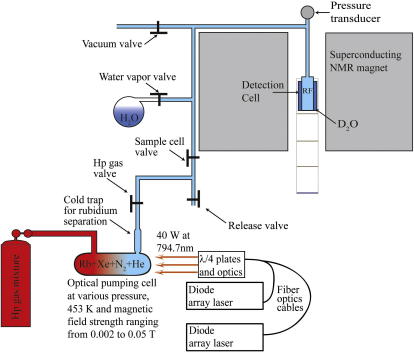
Experimental setup for production and delivery of hp ^131^Xe. Following polarization build-up in the SEOP cell and evacuation of the detection cell, the hp gas valve was opened briefly (with the release valve open and all other valves closed) to release some un-polarized xenon gas from the cold trap region of the pump cell that was not illuminated by the laser. After removal of un-polarized gas, the hp ^131^Xe was then shuttled into the sample cell for detection (hp gas and sample cell valve open, all other valves closed). After detection the vacuum valve was opened (hp gas valve closed) to re-evacuate the detection cell to less than 0.1 kPa. Air-cooled glass wool used to aid in the separation of Rb from the hp gases is not shown but was located in the transfer line near the detection region. Attached to the delivery line between the SEOP cell and the detection cell was a vessel containing only water liquid and H_2_O vapor. The water vapor was transferred into the detection cell for selected experiments as described in the main text. D_2_O shown in the sample region was used for shimming and locking for selected experiments.

For the addition of co-adsorbing water vapor, a vessel filled with 10 ml of liquid water and 3.1 kPa of water vapor was connected to the shuttle system. After the shuttling system was evacuated following the SEOP procedure described in Section 2.1, the water vessel was opened and allowed the system to be filled with water vapor. The vessel was then closed again and delivery of hp ^131^Xe gas was carried out on top of the approximate 3.1 kPa water vapor (see Fig. 1 for details).

### 2.5 Data analysis

*T*_1_ values for hp ^131^Xe were calculated by nonlinear least-squares fitting of the ^131^Xe signal intensity as a function of time and number of applied medium flip angle radio frequency pulses. Since each data point in *T*_1_ measurements was an average of four replicate measurements, the errors reported in this work were calculated as standard deviations. Quadrupolar splittings, 2*ν_Q_*, and linewidths were obtained from ^131^Xe NMR spectra after deconvolution by multi-peak fitting routine using Lorentzian functions. Data analysis and simulations of the polarization curves were performed using Igor Pro, Version 6.11 from Wavemetrics, OR, USA.

## 3 Results and discussion

### 3.1 Separation of hp ^131^Xe from Rb vapor

As detailed in the introduction, spin-exchange optical pumping of ^131^Xe has been explored previously, but these studies focused exclusively on phenomena within the SEOP cells. Although the separation of hp ^3^He, hp ^129^Xe (both spin *I* = 1/2) [5,65,66], and more recently hp ^83^Kr (*I* = 9/2) [64,67–69] from the SEOP alkali metal vapor is well developed, the separation of the hp ^131^Xe from the alkali metal vapor has never been reported. The major obstacle for producing alkali metal free hp ^131^Xe are the large nuclear electric quadrupole interactions found with this isotope. Quadrupolar interactions caused by binary gas-phase collisions [21,26], the formation of gas-phase van der Waals complexes, [24–27], and brief periods of adsorption on surfaces [68] lead to fast longitudinal relaxation that diminishes the level of hyperpolarization. In contrast to ^129^Xe, which has a *T*_1_ time on the order of hours at ambient pressure and temperature [70], a *T*_1_ time below 5 s was observed in this work for gas-phase hp ^131^Xe at a pressure of 120 kPa (using mixture III (93% Xe) at 9.4 T in a 12.6 mm inner diameter glass cell). This value is much shorter than the value of *T*_1_ ≈ 23 s that was expected from the pure gas-phase relaxation given by Eq. (1) [21] because of the relatively large surface to volume ratio in the NMR detection tubes and because of relaxation contributions arising from van der Waals complexes. This fast longitudinal relaxation makes the production and work with hp ^131^Xe more problematic than with hp ^83^Kr, that exhibits *T*_1_ times of around 150 s under similar conditions [25,26]. Despite the larger nuclear electric quadrupole moment of ^83^Kr (*Q* = 25.9 fm^2^) compared to ^131^Xe (*Q* = −11.4 fm^2^) [16], the xenon isotope typically experiences faster quadrupolar driven relaxation under similar conditions due to it’s larger and more easily distortable electron cloud and its smaller nuclear spin value.

**Fig. 2:**
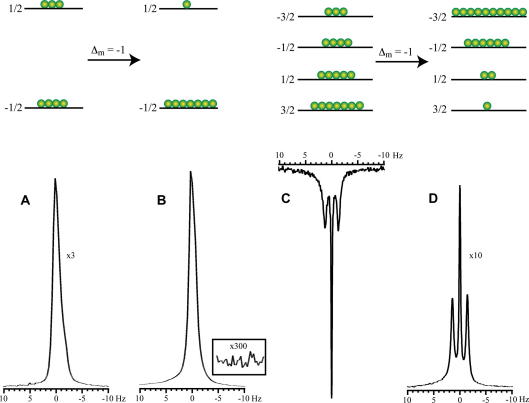
Gas phase NMR spectra collected at 11.7 T of ^129^Xe (A and B) and ^131^Xe (C and D). Also indicated are the associated energy levels at thermal, high temperature equilibrium (A and C) and after optical pumping using the transition *Δ_m_* = −1 (B and D). (A) ^129^Xe NMR spectrum of xenon at 400 kPa (partial pressure) of pure xenon and 100 kPa (partial pressure) oxygen using 250 transients. (B) Hyperpolarized ^129^Xe NMR spectrum with 10 kPa (partial pressure) xenon from a 200 kPa, 5% xenon gas mixture after a single stopped-flow delivery. Note that the scale of (A) is threefold enlarged compared to (B) in this figure. (C) Thermal ^131^Xe NMR spectrum after 1260 transients with a partial pressure of 93 kPa of xenon from a 100 kPa, 93% xenon gas mixture and (D) hyperpolarized ^131^Xe NMR spectrum after a single stopped-flow delivery using 10 kPa xenon partial pressure of a 200 kPa, 5% xenon gas mixture. The scale of (D) is 10-fold enlarged compared to (C). All hyperpolarized spectra are collected with one transient. When correcting for xenon partial pressures and number of transients, enhancements of 33,000 (corresponding to 37% spin polarization) and 1500 (i.e. 0.8% spin polarization) were achieved for ^129^Xe and ^131^Xe, respectively. Note, that up to 2.2% ^131^Xe spin polarization was obtained in later experiments shown in Fig. 4. The differences in the relative phase are due to the positive gyromagnetic ratio of ^131^Xe, as discussed in Section 3.3. All spectra were recorded using xenon with natural abundance isotope distribution.

Because the *T*_1_ for ^131^Xe in the solid phase is extremely short (at 77 K a *T*_1_ slightly above 1 s was observed [17]), freezing the hp-noble gas at liquid nitrogen temperatures – a method frequently used for ^129^Xe separation from the SEOP buffer gases ^4^He and N_2_ [71,72] – would completely destroy the non-equilibrium ^131^Xe polarization. Therefore, cryogenic hp ^131^Xe concentration was not used for any of the experiments described in this work. Rather, the stopped-flow delivery method [64,67–69] depicted in Fig. 1 was applied to efficiently separate the Rb vapor, while avoiding strong depolarization during the gas transfer. The hp ^131^Xe was shuttled after 5–10 min of SEOP through transfer tubing to the pre-evacuated detection cell through pressure-equalization as described in Section 2. Fig. 2 shows the first high field hp ^131^Xe NMR spectrum obtained through stopped-flow SEOP and subsequent rubidium vapor separation.

### 3.2 Linewidth of the ^131^Xe triplet

The spectra of ^131^Xe and ^129^Xe obtained from thermally polarized and hyperpolarized (hp) samples are depicted in Fig. 2. The remarkable appearance of a ^131^Xe triplet in the gas phase is discussed in the introduction and in more detail examined below (see Section 3.6).

The observed linewidth for the ^131^Xe center transition was 0.3 Hz and was approximately constant (deviations < 0.1 Hz) for all the pressures and gas compositions used in this work. A sixfold broader linewidth of 1.8 Hz was observed for the ^129^Xe spectra. A 3.4-fold linewidth ratio is expected from the difference in the gyromagnetic ratios *γ* for the two xenon isotopes if spectral line broadening is dominated by the magnetic field inhomogeneity. Quadrupolar interactions were likely to be responsible for the observed ^131^Xe differential line broadening between the ^131^Xe center transition and the satellite transitions. Unlike the center transition, the linewidth of the ^131^Xe satellite transitions increased with increasing pressure. The satellite transitions shown in Fig. 2D displayed 0.8 Hz and 0.6 Hz linewidths, respectively at higher and lower ppm values.

Differential line broadening can be produced by different relaxation rates for the satellite transition compared to the center transition [73]. However, this would require that the extreme narrowing condition (*τ_c_ω*_0_)^2^ ≪ 1 is violated and thus requires long correlation times $\tau_{c}⩾10^{-9}\;\text{s}$ for ^131^Xe at magnetic fields of 9.4–14 T. The duration of binary, gas-phase collisions is on the order of a few picoseconds, and short-lived Xe–Xe van der Waals molecules have life times of around 10^−10^ s at 1 amagat xenon density [27]. Similarly, the correlation times resulting from surface adsorption are dictated by the average adsorption time, *τ_a_*. The value of *τ_a_* for xenon atoms on glass surfaces at 300 K can be estimated to be ∼10^−10^ s from the expression *τ_a_* = *τ*_0_exp(−*E*/*k_B_T*), where *E* = 0.12 eV is the desorption activation energy xenon on borosilicate glasses [34] and assuming *τ*_0_ = 10^−12^ s. Although none of the correlation times associated with these events are long enough to cause biexponential relaxation, it is possible however that strong xenon adsorption sites are present on the Pyrex surface. The prolonged correlation times at these locations may lead to a violation of the extreme narrowing condition and thus to differential line broadening.

An additional hint for surface interactions as the source for the satellite broadening is the differential broadening between the two satellite transitions. Such differential broadening may be the result of paramagnetic – quadrupolar cross correlation that was observed recently by Jerschow and co-workers by ^23^Na NMR in the presence of paramagnetic contrast agents [74]. The only source for paramagnetism in the sample used for the spectra in Fig. 2 was on the Pyrex surface [75]. Other causes for differential line broadening may be CSA-quadrupolar cross-correlation effects during prolonged surface adsorption. Alternatively, the lineshape may be inhomogeneously broadened by differences in EFG experienced by the xenon atoms in various parts of the container that were not averaged by gas diffusion at the gas pressures used. Although the precise mechanism of the satellite broadening remains speculative thus far, it likely originated from interactions with the Pyrex surface that were scaled down by exchange with the gas phase where the NMR signal was observed. A ‘scaling down’ of surface effects also takes place for quadrupolar splitting that is on the order of 6 MHz on a Pyrex surface [35] but that is observed as a few Hz splitting in the gas phase.

### 3.3 Relative phase and the sign of *γ*

Another distinctive feature shown in Fig. 2 is that thermally polarized ^131^Xe and hyperpolarized ^131^Xe signals were 180° out of phase with respect to each other while both ^129^Xe spectra possessed the same phase. This observation warrants a more detailed explanation. ^131^Xe is unique among the stable (i.e., non-radioactive) noble gas isotopes because it is the only isotope with a positive gyromagnetic ratio *γ*. Therefore, according to *E_m_* = −*γm_z_*ℏ*B*_0_, the energy level *E_m_* with the highest possible positive *z-*quantum number, *m_z_* = +3/2, constitutes the ground state for ^131^Xe. Vice versa, ^3^He, ^21^Ne, ^83^Kr and ^129^Xe have negative gyromagnetic ratios, and the respective ground state is the one with the most negative *m_z_* quantum number. The sign of *γ* determines the sign of the coherence generated by a 90° pulse (H^rf-pulse,x=-γB0I^x) and thus can be important in magnetization transfer or coherence transfer NMR experiments. However the appearance of single-pulse NMR spectra is typically not affected by the sign of *γ* except for a 180° phase difference that is difficult to detect between nuclei with different resonance frequencies. This is different in SEOP experiments since the relative sign of *γ* determines how the energy levels are pumped when using either *σ*^−^ or *σ*^+^ circular polarized light. Therefore, it has consequences even for the outcome of a one-pulse NMR experiments, because the negative *γ* affects the spin population before the radiofrequency-pulse is applied.

This effect is depicted in Fig. 2 where the energy levels and the spin population are sketched for the two isotopes. In SEOP the sign of *Δ_m_* in the nuclear spin transitions depends only on the choice of either *σ*^−^ or *σ*^+^ circular polarized light for the pumping process and is independent of the sign of *γ*. Although the sign of *γ* does not affect *Δ_m_* itself, it still has consequences on the population of the energy levels. For ^129^Xe, the optical pumping transition *Δ_m_ *= −1 pumps the higher energy spin state (*m_z_* = +1/2) down to the lower energy spin state (*m_z_* = −1/2) and thereby causes a reduction in the spin-temperature. In contrast, the same optical pumping transition, *Δ_m_* = −1, pumps low energy spin states in the ^131^Xe system into higher energy spin states leading to an inverted spin population distribution. The phase difference between the thermally polarized spectrum and the hp-spectrum of either isotope is straightforward to compare: when *Δ_m_* = −1 optical pumping was applied, no phase difference was observed for ^129^Xe whereas a 180° relative phase shift was observed for ^131^Xe.

### 3.4 Polarization of spin *I* ⩾ 1/2 nuclei

At high temperature thermal equilibrium (*T* ≫ |*γ*|ℏ*B*_0_/*k_B_*), the polarization *P* of a macroscopic ensemble of separate spins *I* can be described by

(2) $$P=\frac{|\gamma |\hbar B_{0}}{3k_{B}T}(I+1)\text{.}$$

**Fig. 3:**
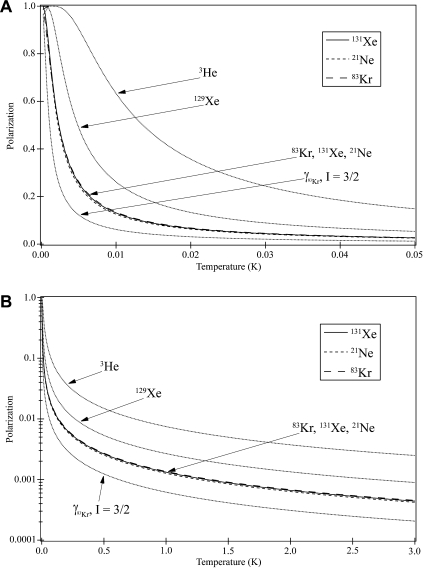
Thermal equilibrium polarization P of spin active noble gas isotopes and a fictitious Kr isotope with spin *I* = 3/2 obtained from Eq. (A2). (A) Thermal equilibrium polarization curves as a function of temperature displayed at temperatures up to 0.05 K. (B) Semi-logarithmic plot of thermal equilibrium polarization curves as a function of temperature extended out to 3 K. To reach 1% polarization for hyperpolarized samples at 300 K and 9.4 T, the following enhancements are needed when compared to thermally polarized samples for each nuclei: 410 for ^3^He; 1121 for ^129^Xe; 2373 for ^21^Ne; 2206 for ^83^Kr; 2270 for ^131^Xe; and 4855 for the fictitious isotope with spin *I* = 3/2 and the same gyromagnetic ratio as ^83^Kr. For the three quadrupolar isotopes (^21^Ne, ^83^Kr, ^131^Xe) nearly identical polarization curves arise.

The maximum possible signal enhancement over the thermal equilibrium at a given field strength and temperature, $f_{\max}^{B_{0}\text{,}T}$, is the inverse of the polarization *P*, assuming ‘Boltzmann-type’ population distribution in the hyperpolarized state. As detailed in the Appendix and demonstrated in Fig. 3, this is true for any temperature or polarization *P* even if Eq. (2) is no longer valid. Fig. 3 shows the thermal polarization *P* obtained through Eq. (A2) [or $(f_{\max}^{B_{0}\text{,}T}){\;}^{-1}$ calculated through Eqs. (A8), (A4), and (A9)] at 9.4 T field strength as a function of the spin temperature *T* for all stable, NMR active noble gas isotopes. Remarkably, the spin temperature dependence of the polarization *P* is almost identical for all three quadrupolar noble gas isotopes. This is not surprising in the case of ^131^Xe and ^21^Ne since both isotopes have the same spin and similar gyromagnetic ratios. However, in the case of ^83^Kr the effect of the smaller gyromagnetic ratio (compared to ^131^Xe and ^21^Ne) is compensated by its higher (*I* = 9/2) spin. For comparison, the behavior of a fictitious spin *I* = 3/2 isotope with the same gyromagnetic ratio as ^83^Kr is also shown in Fig. 3.

The thermal polarization for ^131^Xe at 9.4 T magnetic field strength and 300 K is $P_{{}^{131}\text{Xe}}^{9.4\text{T,}300\text{K}}=4.41\times 10^{-6}$ and therefore a signal enhancement of $f_{\max}^{9.4\text{T,}300\text{K}}=2.27\times 10^{5}$ times the thermal equilibrium signal at 9.4 T and 300 K corresponds to 100% polarization. For comparison, the thermal polarization for ^83^Kr is $P_{{}^{83}\text{Kr}}^{9.4\text{T,}300\text{K}}=4.53\times 10^{-6}$ ($f_{\max}^{9.4\text{T,}300\text{K}}=2.21\times 10^{5}$), and for ^129^Xe is $P_{{}^{129}\text{Xe}}^{9.4\text{T,}300\text{K}}=8.92\times 10^{-6}$ ($f_{\max}^{9.4\text{T,}300\text{K}}=1.12\times 10^{5}$).

### 3.5 Polarization build-up of ^131^Xe

**Fig. 4:**
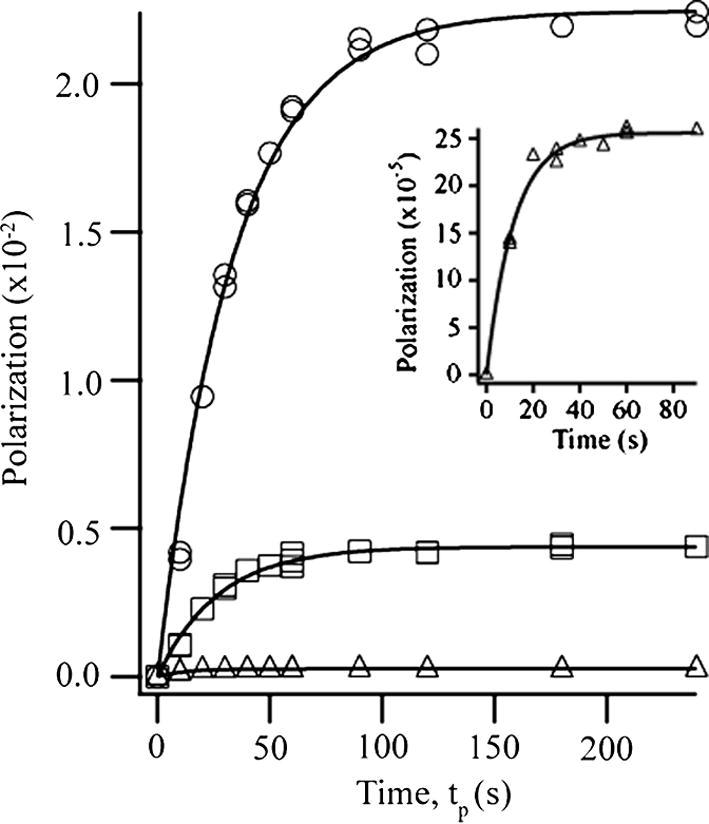
Build-up of hp ^131^Xe spin polarization *P* as a function of SEOP time, *t_p_*, at 2 × 10^−3^ T. Optical pumping was carried out for mixtures I (5% Xe, open circle), II (20% Xe, open square), and III (93% Xe, open triangle) under 40 W laser illumination in a pair of Helmholtz coils. Data were collected at 9.4 T after a stopped-flow delivery cycle of duration *t_p_*. Inset: magnification of short-time polarization build-up for mixture III. The curves are fits of the data to Eq. (4), and the fitting parameter, *A* lead to the following maximum polarization *P* values: mixture I, 2.24 ± 0.03%: mixture II, 0.438 ± 0.007%; and mixture III 0.0256 ± 0.0005%.Values for the rate constant *B* are: mixture I, 0.030 ± 0.001 s^−1^; mixture II, 0.037 ± 0.002 s^−1^; mixture III, 0.085 ± 0.006 s^−1^.

Using the stopped-flow optical pumping method, ^131^Xe signal enhancements on the order of 5000 times greater than thermal signal at *B*_0_ = 9.4 T, 150 kPa, and 297 K were achieved (i.e. approximately 2.2% spin polarization) when mixture I was used. The ^131^Xe polarization build-up reached a steady-state relatively quickly compared to other noble gas isotopes (^3^He, ^129^Xe and, ^83^Kr – at similar SEOP conditions). The time dependence for the hp ^131^Xe polarization build-up is shown in Fig. 4 for the three different mixtures (5%, 20% and 93% Xe) under 40 W of *σ*^−^ circularly polarized 794.7 nm laser light. To monitor the ^131^Xe polarization build-up, the magnetic field at the SEOP cell was initially switched off, while the cell was maintained under constant laser illumination at a constant temperature (453 K) and pressure (150 kPa) for 5–10 min. This procedure produced a ‘starting point’ at stable SEOP conditions but with no hyperpolarized ^131^Xe present and allowed for regeneration of the rubidium vapor after the shuttling procedure. The magnetic field of a pair of Helmholtz coils was then turned on for incremented time period, *t_p_*, after which the hp ^131^Xe was transferred to the sample cell where it was detected. The polarization value was obtained from the hp ^131^Xe signal intensities through comparison to the thermal signal of ^131^Xe described in the experimental section.

The time dependent build-up of hyperpolarization is described as [72]:

(3) $$P_{{}^{131}\mathrm{Xe}}^{\text{SEOP}}=\frac{\gamma_{\mathrm{se}}}{\gamma_{\mathrm{se}}+\Gamma }\cdot \frac{\gamma_{\mathrm{op}}}{\gamma_{\mathrm{op}}+\underset{i}{\sum }\kappa_{\mathrm{sd}}^{i}[M_{i}]}(1-e^{-(\gamma_{\mathrm{se}}+\Gamma )t_{p}})\text{,}$$

where *γ_se_* is the Rb–Xe spin exchange rate and *Γ* = 1/*T*_1_ is the quadrupolar driven fast self-relaxation rate of ^131^Xe. The destruction of Rb spin polarization by collisions with inert gas atoms is described by the sum of the products of the rate constants, $\kappa_{\mathrm{sd}}^{i}$, with their corresponding gas atom number densities [*M_i_*]. The optical pumping rate per Rb atom, *γ_op_*, depends on experimental parameters such as laser power, SEOP cell design, and SEOP temperature that were kept constant for all build-up experiments reported here. However only a reduced form of Eq. (3) was used for fitting of the experimental data since *γ_se_* and *Γ* were unknown under the SEOP conditions used in this work:

(4) $$P_{{}^{131}\mathrm{Xe}}^{\text{SEOP}}(t)=A(1-e^{-{\mathrm{Bt}}_{p}})\text{.}$$

The lower the xenon concentration used in the gas mixture, the larger was the resulting pre-exponential parameter *A*. The steady-state polarization $P_{{}^{131}\mathrm{Xe}}^{\text{SEOP}}(\max)$ (i.e. at infinite long SEOP times) determined through *A* was 2.24 ± 0.03 for mixture I (5% xenon), 0.438 ± 0.007 for mixture II (20% xenon), and 0.0256 ± 0.0005 for mixture III (93% xenon). The ratios between the values obtained for *A* were 1:0.20:0.011 for mixture I, II, and III respectively. A strong polarization dependence on the xenon density [Xe] is expected from Eq. (3) and from the large rubidium depolarization rate constant $\kappa_{\mathrm{sd}}^{\text{Xe}}=5.2\times 10^{-15}\;{\text{cm}}^{3}\;{\text{s}}^{-1}$ for xenon [72,76]. The strong polarization dependence on [Xe] is well known for ^129^Xe SEOP, however the approximately 100-fold reduction of the ^131^Xe polarization between mixtures I to III exceeds significantly the effect previously observed with SEOP of the spin *I *= 1/2 isotope [77]. If the xenon self relaxation *Γ* is omitted in Eq. (3) and if one neglects the effects of nitrogen and helium (note that $\kappa_{\mathrm{sd}}^{\text{He}}:\kappa_{\mathrm{sd}}^{{\text{N}}_{2}}:\kappa_{\mathrm{sd}}^{\text{Xe}}\approx 3.8\times 10^{-4}:1.7\times 10^{-3}:1$) [72,76], the steady-state polarization reached after long SEOP times is described by $P_{{}^{131}\mathrm{Xe}}^{\text{SEOP}}(\max)=\gamma_{\mathrm{op}}/(\gamma_{\mathrm{op}}+\kappa_{\mathrm{sd}}^{\text{Xe}}[\text{Xe}])$. For $\kappa_{\mathrm{sd}}^{\text{Xe}}[\text{Xe}]\gg \gamma_{\mathrm{op}}$, the dependence upon the xenon density is $P_{{}^{131}\mathrm{Xe}}^{\text{SEOP}}(\max)\propto [\text{Xe}]{\;}^{-1}$.

This proportionality describes approximately the observations of previous work with ^129^Xe SEOP [77], where the same laser and similar SEOP cells had been used under continuous flow conditions. It was found that $\kappa_{\mathrm{sd}}^{\text{Xe}}[\text{Xe}]$ exceeds *γ_op_* by about one order of magnitude. For the mixtures I, II and III one would therefore expect a ratio for *A* of 1:0.25:0.054, i.e. an approximately 20-fold reduction in polarization between I and III. The 100-fold reduction found with ^131^Xe suggest that, in contrast to ^129^Xe, the relaxation rate constant *Γ* in Eq. (3) cannot be neglected for ^131^Xe in mixture III. The term *γ_se_*/(*γ_se_* + *Γ*) contributes roughly with a factor of five to the polarization difference between mixtures III and I, while it contributes relatively little to the polarization difference between mixtures II and I.

The value for *Γ* can be estimated from Eq. (1) and increases approximately 18 times from 0.18 × 10^−2^ s^−1^, to 0.72 × 10^−2^ s^−1^, and to 3.3 × 10^−2^ s^−1^ for mixture I, II and III respectively, at the xenon density found at 150 kPa total pressure and 453 K SEOP temperature. However, the contributions from the other gases to the ^131^Xe relaxation are neglected. Previous work with hp ^83^Kr spectroscopy [26] has shown that other inert gases contribute quite substantially to the observed relaxation, but the estimate made above is probably reasonable for mixture III due to its high xenon concentration. There are however further problems: Eq. (1) is valid for *T* = 298 K only [23] and in addition the relaxation will be affected by the wall relaxation and by van der Waals complexes in the gas phase [25]. Nevertheless, the values above, in particular for mixture III, will be used for some further considerations.

The spin exchange rate *γ_se_* is a function of xenon density dependent term and a xenon density independent term [78]:

(5) $$\gamma_{\mathrm{se}}=[\text{Rb}]\left(\frac{\gamma_{\text{RbXe}}}{\left[\text{Xe}\right]}+\langle \sigma v\rangle \right)$$

were the rate constant *γ*_RbXe_ describes xenon spin exchange during Rb–Xe van der Waals complexes and 〈*σv*〉 is the spin exchange cross section for binary collisions. The precise values of *γ*_RbXe_ and 〈*σv*〉 are uncertain for ^131^Xe under the SEOP conditions used in this work. However, Eq. (5) states that *γ_se_* is reduced with increasing xenon density until it assumes the form *γ_se_* = [Rb]〈*σv*〉, while Eq. (1) states that *Γ* increases with increasing xenon density. As stated above, the term *γ_se_*/(*γ_se_* + *Γ*) in Eq. (3) does not seem to contribute substantially to the polarization change between mixtures I and II but contributes with a fivefold reduction in the expected polarization between mixture I and III. It can be concluded that *Γ* > *γ_se_* at xenon partial pressures somewhere above 30 kPa (i.e. mixture II at 150 kPa total pressure).

Based on the observations and assumptions made above, one can conclude that for mixture III *γ_se_*/(*γ_se_* + *Γ*) ≈ 0.2 and hence *Γ* ≈ 4*γ_se_*. From the fitting parameter *B* = *γ_se_* + *Γ* that was determined as (8.5 ± 0.6) × 10^−2^ s^−1^ for mixture III one can conclude that *γ_se_* ≈ 1.7 × 10^−2^ s^−1^ and estimate *Γ* ≈ 6.8 × 10^−2^ s^−1^ for the 93% xenon mixture. This *Γ* value is about twice as large as the rate constant $T_{1}^{-1}\approx 3.3\times 10^{-2}\;{\text{s}}^{-1}$ expected form Eq. (1). However, an increase of the ^131^Xe *T*_1_ relaxation by a factor of two due to surface contributions and van der Waals complexes in the pump cell is not unreasonable, as can be illustrated by the following estimate: In the Section 3.1 a ^131^Xe *T*_1_ ≈ 5 s in the 12.6 mm inner diameter NMR tube was found. From the simplified expression $T_{1}^{-1}=T_{1}(\mathrm{gas}){\;}^{-1}+T_{1}(\mathrm{surface}){\;}^{-1}$ one obtains *T*_1_(*surface*)^−1^ ≈ 16 × 10^−2^ s^−1^ for this NMR tube neglecting contributions from van der Waals complexes. This value is too high but the relaxation time due to surface interactions scales directly with the surface to volume ratio [64] and the (uncoated) pump cell has a 27 mm inner diameter leading to *T*_1_(*surface*)^−1^ ≈ 8 × 10^−2^ s^−1^ – a value close to that for *Γ* found above. In addition, the ^131^Xe surface contribution to the relaxation is expected to be further reduced by the elevated temperature [67] and by the presence of rubidium metal [32].

**Fig. 5:**
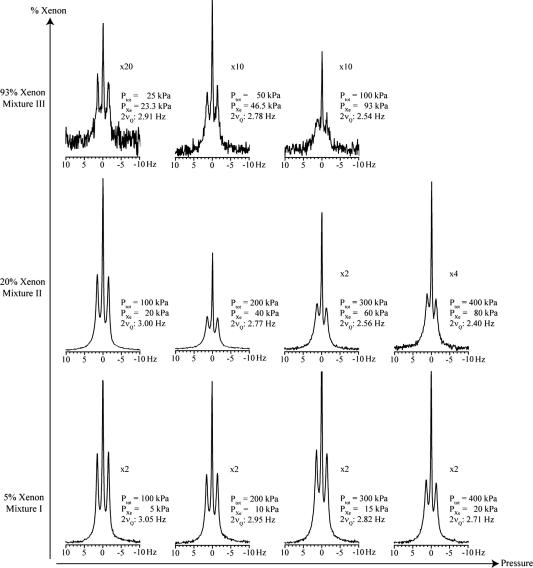
Collection of hp ^131^Xe gas phase NMR spectra at various gas compositions (i.e. xenon partial pressures) and total gas pressures. Hp ^131^Xe gas was contained in an 8 mm (6.5 mm inner diameter) plain NMR tube at 11.7 T field strength and 289 K. Magnification factors, total pressure (*P_tot_*), partial pressure of xenon (*P*_Xe_) and quadrupolar splitting (2*ν_Q_*) are presented beside each spectrum. Spectra with identical xenon concentration are grouped within a row. For each row, the total pressure increases from the left to the right.

In summary, ^131^Xe polarization is strongly dependent on the xenon density, most significantly due to rubidium depolarization. However, the ^131^Xe polarization is further affected by the xenon density dependent quadrupolar relaxation. The consequences of the combined effects is that high density SEOP is even more inefficient for ^131^Xe than for ^129^Xe. This inefficiency is illustrated in Fig. 5 where a distinct decrease in optical pumping efficiency was observed in mixture II and mixture III as the pressure was increased. At 100 kPa pressure used for these experiments only 0.03% polarization was generated with mixture III, and the signal was barely observable at higher pressures. However, at the lowest xenon concentration (mixture I), the applied pressure had a negligible effect on the SEOP conditions. The likely source for the pressure independence was that the polarization diminishing effect at increasing pressure was compensated by the competing effect of the increasing SEOP efficiency due to improvement of *γ_op_* that can be caused by the broadening of the rubidium adsorption linewidth [72,76,77].

### 3.6 Influence of pressure and concentration on quadrupolar splitting of ^131^Xe

A dependence of the quadrupolar splitting on both the total pressure of the sample and the gas composition was observed with hp ^131^Xe at 11.7 T. In Fig. 5 the hp ^131^Xe spectra are shown for mixtures I and II (5% and 20% xenon, respectively) with pressures ranging from 100 to 400 kPa and for mixture III (93% xenon) with pressures ranging from 25 to 100 kPa. Hp spectra for mixture III at pressures higher than 100 kPa were not recorded due to the low spin polarization obtained at these conditions.

The quadrupolar splitting varies from the smallest observed value of 2.40 Hz at 400 kPa in mixture II to the largest value of 3.05 Hz at 100 kPa of mixture I. The quadrupolar splitting of ^131^Xe observed in mixture I decreased slightly over the pressure range of 100–400 kPa. At 100 kPa the quadrupolar splitting is 3.05 Hz and it decreased to 2.71 Hz at 400 kPa, a change of 0.34 Hz. Mixture II showed a greater decrease in quadrupolar splitting than was observed in mixture I over the same pressure range. The quadrupolar splitting was 3.00 Hz at 100 kPa and 2.40 Hz at 400 kPa, for an overall change of 0.60 Hz, almost double the change observed in mixture I. The quadrupolar splitting observed in mixture III decreased from 2.91 Hz at 25 kPa to 2.54 Hz at 100 kPa, a change of 0.37 Hz over the pressure range.

A pressure dependence of the ^131^Xe quadrupolar splitting was predicted in earlier work considering much lower xenon densities, in particular with respect to the xenon free path length λ and the xenon diffusion, that are not applicable at the pressures used in this work [31]. Later experimental work found no influence of the nitrogen buffer gas partial pressure between 2.6 kPa and 32 kPa on the ^131^Xe quadrupolar splitting [32]. The pressure dependence of the ^131^Xe spectra observed in Fig. 5 may have been caused by changes in quadrupolar splitting arising from the interactions with the glass surface. Noble gases at ambient temperature will exhibit a very low surface coverage rate *θ* that is dependent on xenon density [Xe] as described by the Henry isotherm. This would predict a constant *θ*/[Xe] and hence alternating xenon densities should not have affected the splitting observed in the gas phase. However, this picture would change in the presence of strong xenon adsorption sites caused by defects on the surface that may experience xenon coverage rates close to saturation at the pressure used in this work. The relative contribution of these sites to the observed quadrupolar splitting would be reduced with increasing pressure. As noted above, the presence of strong adsorption sites also may be a possible explanation of the observed differential line broadening.

### 3.7 The effect of H_2_O vapor on ^131^Xe relaxation and quadrupolar splitting

**Fig. 6:**
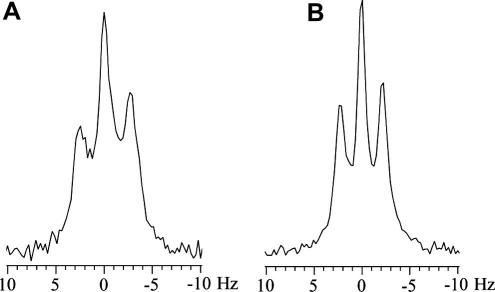
Hp ^131^Xe NMR spectra collected from a 5 mm (4.1 mm inner diameter) plain NMR tube (A) in the absence of water vapor and (B) with 3.1 kPa of H_2_O. Both spectra were collected at a pressure of 100 kPa of mixture I (5% xenon) at 14.1 T with ^131^Xe frequency of 49.47 Hz. (A) The measured quadrupolar splitting was 5.24 Hz. (B) The measured quadrupolar splitting was 4.46 Hz under conditions identical to (A) except for the admission of water vapor (3.1 kPa) prior to hp ^131^Xe delivery.

The addition of co-adsorbing molecules was used to demonstrate that the gas phase quadrupolar splitting is indeed influenced by changing surface interactions. The ^131^Xe quadrupolar splitting observed at 14.1 T in a 5 mm NMR tube at 100 kPa and 290 K without the presence of water vapor was 5.24 Hz as shown in Fig. 6A. Upon the addition of 3.1 kPa of water vapor as described in the experimental section, the splitting was reduced to 4.46 Hz as shown in Fig. 6B. The effect of the water vapor was completely reversible as demonstrated by evacuating the NMR tube and flushing with dry nitrogen at least three times. Following this treatment, quadrupolar splittings within 0.2 Hz of the values obtained prior to addition of water vapor were observed.

The reduced surface interactions of xenon in the presence of water vapor also affects the ^131^Xe relaxation times. It was previously shown that the adsorption of water onto an aerogel surface changes the ^131^Xe spin–spin (*T*_2_) relaxation, an effect that was used for surface sensitive MRI contrast with liquefied xenon [51]. In the current work, a *T*_1_ relaxation time increase in the presence of water vapor was found using gas-phase hp ^131^Xe contained in a Pyrex container. The three gas mixtures (I, II, and III) were optically pumped and spin–lattice relaxation times for each mixture were collected in a 15 mm outer diameter Pyrex sample tube at a field strength of 9.4 T and a temperature of 290 K. These data are presented in Table 1 and demonstrated the reduced ^131^Xe relaxation in the presence of water vapor with a relaxation time of *T*_1_ = 14.0 ± 0.2 s that was increased by about 40% compared to the dry gas mixture with *T*_1_ = 9.9 ± 0.1 s. The effect of water vapor on ^83^Kr relaxation was previously demonstrated to have a similar tendency as was observed with ^131^Xe in this work [67,69].

## 4 Conclusion

Alkali metal vapor free hp ^131^Xe was generated with a signal enhancement up of 5000 times the thermal equilibrium polarization at 9.4 T field strength and ambient temperatures for dilute xenon mixture. The maximum ^131^Xe enhancement obtained in this work corresponded to 2.2% spin polarization. Like in spin *I* = 1/2 systems, the polarization of hp-noble gases with spin *I* > 1/2 can be calculated by simple multiplication of the thermal high temperature polarization with the enhancement factor of the hp signal over the thermal high temperature NMR signal. A general equation was derived (Eq. (2), see Appendix for details) to describe the thermal spin polarization *P* at high temperatures for nuclei with any spin *I* value. Because of its positive gyromagnetic ratio, unique for ^131^Xe among all stable noble gas isotopes, the relative phase difference between thermal signal and hp signal is 180° opposite to that of any other noble gas isotope.

The time dependence of the polarization build-up accelerated, and the maximum polarization value decreased, with increasing xenon partial pressure. Because of xenon partial pressure dependent quadrupolar relaxation, this effect is more pronounced at higher xenon density for ^131^Xe SEOP than for ^129^Xe SEOP. The obtained hp ^131^Xe signals displayed a quadrupolar splitting that is known to be magnetic field – and surface interaction dependent. In this work, an additional xenon partial pressure dependence upon the splitting was found. A possible explanation may be the effects arising from strong adsorption sites on the surface that may also be responsible for the observed differential line broadening between center and satellite transitions. Finally, alkali metal vapor free hp ^131^Xe allowed for experiments with co-adsorbing water molecules on the surface. It was found that the presence of water vapor significantly reduces the observed ^131^Xe quadrupolar splitting and prolongs the ^131^Xe *T*_1_ relaxation times.

The quadrupolar splitting in the gas phase is uniquely observed thus far with ^131^Xe NMR spectroscopy. The disagreement in earlier theoretical work makes the experimental study of the magnetic field dependent contribution to the quadrupolar splitting important. The investigation of this effect is complicated by surface interactions and by the newly found xenon partial pressure dependence of the quadrupolar splitting. Hp ^131^Xe may provide better insights into the surface relaxation processes including those that produce higher rank tensor elements [48] and that may interfere with the observed coherent processes [37,48].

The fast ^131^Xe *T*_1_ relaxation in porous media makes widespread applications of hp ^131^Xe NMR spectroscopy and imaging unlikely. However, hp ^131^Xe may help to provide insights into another probe system, i.e. hp ^83^Kr (*I *= 9/2), that has recently been explored as a new MRI contrast agent with potential applications for pulmonary studies [68,69,79,80]. Finally, hp ^131^Xe can be used to study xenon van der Waals complex formation in the gas phase that are also important for hp ^129^Xe. Such processes are difficult to study with ^129^Xe because of its extremely slow relaxation [27]. Pure gas phase ^131^Xe faster relaxation times (on the order of tens of seconds) will allow for thorough studies of various pressures and mixtures.

## Figures and Tables

**Table 1:** *T*_1_ values for ^131^Xe with various gas compositions measured for three different concentrations of xenon, nitrogen and helium at 140 kPa and 9.4 T. The column labeled ‘Hydrated glass tube’ refers to the experiment with water vapor as co-adsorbing molecules. The values reported are the mean and standard deviation of four replicate *T*_1_ measurements.

Dehydrated	Dehydrated	Dehydrated	Hydrated
Glass tube	Glass tube	Glass tube	Glass tube
5% Xe	20% Xe	93% Xe	20% Xe
19.3 ± 0.3 s	9.9 ± 0.1 s	4.7 ± 0.1 s	14.0 ± 0.2 s

## Appendix A

A general definition of the nuclear spin polarization *P* is given by Wenckebach [81]:

(A1) P=1ℏI|Tr{ρI^z}|,

which is written here in slightly different form where ${\overset{ˆ}{I}}_{z}$ is the spin operator, ℏ is the Planck constant *h*/2*π*, and *ρ* is the normalized density matrix. The polarization *P* in Eq. (A1) is obtained from the magnetization |*M_z_*| normalized through its maximum possible value of $|M_{z}|=\hbar |\gamma |I$ at the theoretical limit of *T *= 0 K.

Eq. (A1) is rewritten as:

(A2) $$P^{B_{0}\text{,}T}=\frac{\left|\sum_{m_{z}=-I}^{+I}(m_{z}\cdot e^{m_{z}\gamma \hbar B_{0}/k_{B}T})\right|}{I\cdot \sum_{m_{z}=-I}^{+I}e^{m_{z}\gamma \hbar B_{0}/k_{B}T}}$$

with *m_z_* as the *z*-quantum number of spin *I*, *γ* as the gyromagnetic ratio, ℏ as the Planck constant *h*/2*π*, and *k_B_* as the Boltzman constant.

For a temperature *T* ≫ |*γ*|ℏ*B*_0_/*k_B_*, the polarization simplifies to:

(A3) $$P^{B_{0}\text{,}T\gg |\gamma |\hbar B_{0}/k_{B}}=\frac{|\gamma |\hbar B_{0}}{3k_{B}T}(I+1)$$

The polarization *P* of a thermally polarized spin I⩾1/2 system can be readily be calculated using Eq. (A3) that transforms into the well-known equation $P_{I=1/2}^{B_{0}\text{,}T}=|\gamma |\hbar B_{0}/2k_{B}T$ for spin *I* = 1/2 systems. However, the definition of polarization *P* is only useful if *P* is linearly dependent upon the observed signal intensity at any temperature. For instance, this is not the case if one defines the spin polarization of a spin *I* > 1 systems through the sum of the population differences between two adjacent energy levels [80].

The signal intensity $S_{\mathrm{tp}}^{B_{0}\text{,}T}$ caused by thermal polarization (i.e. Boltzmann polarization) of the sample at temperature *T* and magnetic field *B*_0_ is determined by:

(A4) $$S_{\mathrm{tp}}^{B_{0}\text{,}T}=A\gamma^{2}B_{0}\frac{\sum_{m_{z}=-I}^{I}(C_{I\text{,}m_{z}}^{+}){\;}^{2}(e^{(m_{z}+1)\gamma \hbar B_{0}/k_{B}T}-e^{m_{z}\gamma \hbar B_{0}/k_{B}T})}{\sum_{m_{z}=-I}^{I}e^{\gamma m_{z}\hbar B_{0}/k_{B}T}}\text{.}$$

Note that Eq. (A4) describes total integrated signal intensity – i.e. obtained from the sum of all integrated peaks if a spitting is present. Eq. (A4) is allowed to dependent on the sign of the gyromagnetic ratio in order to accommodate the absolute phase of the signal. The transition matrix elements,

(A5) (CI,mz+) 2=|I,mz+1I^+I,mz|2,

are obtained from

(A6) I^+I,mz=ℏI(I+1)-mz(mz+1)I,mz+1.

All contributions to the signal intensity other than *γ*, *B*_0_, and the populations of the various quantum states are combined in the coefficient *A*. At ambient temperatures (i.e. *T* ≫ |*γ*|ℏ*B*_0_/*k_B_*), Eq. (A4) can be simplified to:

(A7) $$S_{\mathrm{tp}}^{B_{0}\text{,}T\gg |\gamma |\hbar B_{0}/k_{B}}=\frac{2}{3}\frac{A\gamma^{3}\hbar^{3}B_{0}^{2}}{k_{B}T}\cdot I(I+1)\text{,}$$

where the intensity has been allowed once again to be phase sensitive to the sign of *γ*. Using the signal intensity $S_{\mathrm{tp}}^{B_{0}\text{,}T}$ from Eqs. (A4), (A7), the maximum enhancement can be defined as:

(A8) $$f_{\max}^{B_{0}\text{,}T}=(S_{\mathrm{tp}}^{B_{0}\text{,}T\to 0\text{K}}/S_{\mathrm{tp}}^{B_{0}\text{,}T}\text{,}$$

where $S_{\mathrm{tp}}^{B_{0}\text{,}T\to 0\text{K}}$ is the maximum possible (thermal) signal intensity expected at *T = *0 K (phase sensitive):

(A9) $$S_{\mathrm{tp}}^{B_{0}\text{,}T\to 0\text{K}}=A\frac{\gamma^{3}}{\left|\gamma \right|}B_{0}(C_{I\text{,}m_{z}=-I}^{+}){\;}^{2}=A\frac{\gamma^{3}}{\left|\gamma \right|}\hbar^{2}B_{0}2I$$

Using the Eqs. (A7)–(A9), the maximum enhancement factor from hyperpolarization (assuming a non-thermal equilibrium, but still Boltzmann type of population distribution) compared to a thermal signal at ambient temperature is [80]:

(A10) $$f_{\max}^{B_{0}\text{,}T\gg |\gamma |\hbar B_{0}/k_{B}}=\frac{3k_{B}T}{\left|\gamma |\hbar B_{0}(I+1\right)}\text{.}$$

The maximum enhancement factor $f_{\max}^{B_{0}\text{,}T}$ in Eq. (A10) is the inverse of the polarization *P* at high temperatures given in Eq. (A3). Further, $f_{\max}^{B_{0}\text{,}T}$ in Eq. (A8) (inserting Eqs. (A4) and (A9)) is the inverse to the polarization *P* in Eq. (A2) at any temperature. As a practical consequence, the polarization *P* of any NMR active hyperpolarized system can be calculated from the polarization of the thermally polarized system using Eq. (A3) multiplied with the enhancement factor of the hp signal over the thermal signal.
